# Supplementary material for: Iterative improvement in the automatic modular design of robot swarms
Source: PeerJ Comput Sci. 2020 Dec 7;6:e322. doi: 10.7717/peerj-cs.322 (PMC7924708; doi:10.7717/peerj-cs.322)
Supplement: Supplemental Information 3 [file peerj-cs-06-322-s003.zip › argos3/doc/api/standalone/a00498_source.html]

ARGoS: plugins/robots/generic/control\_interface/ci\_ground\_sensor.cpp Source File


- Main Page
- Related Pages
- Namespaces
- Classes
- Files

- File List
- File Members

# plugins/robots/generic/control\_interface/ci\_ground\_sensor.cpp

Go to the documentation of this file.

```
00001 
00007 #include "ci_ground_sensor.h"
00008 
00009 #ifdef ARGOS_WITH_LUA
00010 #include <argos3/core/wrappers/lua/lua_utility.h>
00011 #endif
00012 
00013 namespace argos {
00014 
00015    /****************************************/
00016    /****************************************/
00017 
00018 #ifdef ARGOS_WITH_LUA
00019    void CCI_GroundSensor::CreateLuaState(lua_State* pt_lua_state) {
00020       CLuaUtility::StartTable(pt_lua_state, "ground");
00021       for(size_t i = 0; i < m_tReadings.size(); ++i) {
00022          CLuaUtility::AddToTable(pt_lua_state, i+1, m_tReadings[i]);
00023       }
00024       CLuaUtility::EndTable(pt_lua_state);
00025    }
00026 #endif
00027 
00028    /****************************************/
00029    /****************************************/
00030 
00031 #ifdef ARGOS_WITH_LUA
00032    void CCI_GroundSensor::ReadingsToLuaState(lua_State* pt_lua_state) {
00033       lua_getfield(pt_lua_state, -1, "ground");
00034       for(size_t i = 0; i < m_tReadings.size(); ++i) {
00035          lua_pushnumber(pt_lua_state, i+1           );
00036          lua_pushnumber(pt_lua_state, m_tReadings[i]);
00037          lua_settable  (pt_lua_state, -3            );
00038       }
00039       lua_pop(pt_lua_state, 1);
00040    }
00041 #endif
00042 
00043 
00044    /****************************************/
00045    /****************************************/
00046 
00047    const std::vector<Real>& CCI_GroundSensor::GetReadings() const {
00048      return m_tReadings;
00049    }
00050 
00051    /****************************************/
00052    /****************************************/
00053 
00054 }
```

---

Generated on 10 Jul 2018 for ARGoS by 
 1.6.1
